# The traffic ATPase PilF interacts with the inner membrane platform of the DNA translocator and type IV pili from *Thermus thermophilus*

**DOI:** 10.1002/2211-5463.12548

**Published:** 2018-11-26

**Authors:** Kerstin Kruse, Ralf Salzer, Beate Averhoff

**Affiliations:** ^1^ Molecular Microbiology & Bioenergetics Institute of Molecular Biosciences Goethe University Frankfurt Germany; ^2^ Present address: Structural Studies Division Medical Research Council – Laboratory of Molecular Biology Cambridge Biomedical Campus, Francis Crick Ave Cambridge CB2 OQH UK

**Keywords:** ATPase, DNA transporter, natural competence, PilF, thermophile, type IV pili

## Abstract

A major driving force for the adaptation of bacteria to changing environments is the uptake of naked DNA from the environment by natural transformation, which allows the acquisition of new capabilities. Uptake of the high molecular weight DNA is mediated by a complex transport machinery that spans the entire cell periphery. This DNA translocator catalyzes the binding and splitting of double‐stranded DNA and translocation of single‐stranded DNA into the cytoplasm, where it is recombined with the chromosome. The thermophilic bacterium *Thermus thermophilus* exhibits the highest transformation frequencies reported and is a model system to analyze the structure and function of this macromolecular transport machinery. Transport activity is powered by the traffic ATPase PilF, a soluble protein that forms hexameric complexes. Here, we demonstrate that PilF physically binds to an inner membrane assembly platform of the DNA translocator, comprising PilMNO, via the ATP‐binding protein PilM. Binding to PilMNO or PilMN stimulates the ATPase activity of PilF ~ 2‐fold, whereas there is no stimulation when binding to PilM or PilN alone. A PilM_K26A_ variant defective in ATP binding still binds PilF and, together with PilN, stimulates PilF‐mediated ATPase activity. PilF is unique in having three conserved GSPII (general secretory pathway II) domains (A–C) at its N terminus. Deletion analyses revealed that none of the GSPII domains is essential for binding PilMN, but GSPIIC is essential for PilMN‐mediated stimulation of ATP hydrolysis by PilF. Our data suggest that PilM is a coupling protein that physically and functionally connects the soluble motor ATPase PilF to the DNA translocator via the PilMNO assembly platform.

**Abbreviations**
DDM: *n*‐dodecyl β‐d‐maltopyranoside
GSPII: general secretory pathway II protein E, N‐terminal domain
T2SS: type II secretion system
T4P: type IV pilus

The acquisition of novel genetic determinants facilitates microorganisms to colonize diverse environmental niches and is as a major force in adaptation and diversification. Uptake of free DNA via natural transformation is mediated by a macromolecular machinery that binds DNA at the cell surface, transports double‐stranded DNA to the cytoplasmic membrane, cleaves the double strand into single‐stranded DNA, and translocates the single strand across the cytoplasmic membrane into the cytoplasm 1. Natural transformation systems are widespread in bacteria and are related to type IV pili (T4P) and type II secretion systems (T2SS) 1, 2, 3, 4, 5. The macromolecular DNA transporter comprises highly conserved core components such as pilins/pseudopilins, assembly ATPases powering the assembly of the pseudopilins/pilins, a soluble PilM protein with an actin‐like fold, components associated with the inner membrane assembly platform, such as PilC, PilN, and PilO, and secretins which form multimeric complexes for the import of DNA and extrusion of T4P through the outer membrane 1, 5, 6, 7. However, information with respect to the organization of the complex DNA transporter and molecular details of interactions and assembly of the subunits are limited.

To get insights into the structure and function of natural transformation machineries, we have chosen *Thermus thermophilus*, a Gram‐negative, thermophilic bacterium, which exhibits the highest natural transformation frequencies known to date 8. The DNA translocator of *T. thermophilus* contains at least 16 components, many of them playing a dual role in T4P biogenesis 9, 10, 11.

A secretin complex, PilQ, guiding the DNA through the outer membrane comprises of a stable cone and a cup structure and six ring structures with a large central channel 12, 13. The PilQ complex is highly dynamic and undergoes major conformational changes by opening and closing two periplasmic gates 14. PilQ of *T. thermophilus* differs from all known secretins, which contain only 2–4 rings and only one gate 15, 16, 17. The elongation of the PilQ complex might be an adaptation to the wide periplasm of *T. thermophilus* 14. Localization of PilQ in the outer membrane strongly depends on the unique outer membrane protein PilW 18, which has no homologs in other organisms. The *pilW* gene is located in the genomic locus that in other systems is occupied by *pilP*, which encodes for a lipoprotein not found in *T. thermophilus* 19, 20, 21. The PilQ complex binds the DNA in the first place and is suggested to be the counter bearing for a pilus‐like DNA translocator rod or the T4P made by distinct pilin subunits 10. A subcomplex in the cytoplasmic membrane forms the base for retraction and extension of the pilus structures comprising the major pilin PilA4 and the DNA translocator pseudopilus comprising PilA4 and the minor pilins PilA1–PilA3. In addition, the membrane proteins PilC, PilN, and PilO and the soluble but membrane‐associated protein PilM are essential for the functionality and/or biogenesis of the DNA transporter and T4P 9. Apart from that, the inner membrane base also provides the translocation machinery for the single‐stranded DNA that is bound in the periplasm by a membrane‐anchored DNA binding protein designated ComEA 22, 23. Transport through the inner membrane is suggested to be mediated by an inner membrane channel formed by ComEC 24. The traffic ATPase PilF, which was found to form zinc binding hexameric complexes, provides the driving force of the DNA translocator and T4P assembly 11, 25, 26. PilF has been shown to form a dumbbell‐like structure with two elongated stacked rings, one of which is formed by the C‐terminal ATPase domains and the other by the N‐terminal domains 27, 28. PilF of *T. thermophilus* has an unusually long N terminus with three ‘general secretory pathway II protein E, N‐terminal domains’ (GSPII), whereas all other known traffic ATPases only contain one GSPII domain 25, 29, 30, 31. This triplication in *T. thermophilus* might lead to higher complex stability which allows for purification of hexamers without the use of assisting protein fusions as described for purifications of other traffic ATPase complexes such as GspE from *Vibrio cholerae* or PilB from *Myxococcus xanthus* 32, 33.

Subcellular localization studies of the DNA transporter proteins PilM, PilN, and PilO from *T. thermophilus* provided clear evidence that these components are part of the inner membrane assembly platform 18. Structural analyses revealed that *T. thermophilus* PilM binds ATP and interacts with the N terminus of PilN. The latter was suggested to affect the ATP‐binding site of PilM 34. Co‐expression of PilM, PilN, and PilO led to the identification of PilMNO complexes 35. These subcomplexes of the DNA translocator were found to bind the major pilin PilA4. Electron microscopy 3D reconstructions of PilMN, PilMNO, and PilMNO bound to PilA4 suggest that PilN drives dimerization of the PilMN complex, followed by binding of two PilO monomers causing the dissociation of PilN periplasmic domains. The latter is suggested to allow binding of the pilin PilA4 to the periplasmic domains of PilN and PilO 35. However, despite the broad analyses of the DNA transporter of *T. thermophilus* the key question remains how the inner membrane platform of the DNA translocator is connected to the energy providing traffic ATPase PilF.

The conserved PilM, PilN, and PilO proteins of different T4P systems have been shown to interact with each other 21, 36, 37, 38, 39, 40, 41, 42. Moreover, PilM of the T4P systems of *M. xanthus*,* Pseudomonas aeruginosa*, and *Neisseria meningitidis* were found to interact with the traffic ATPase PilB/PilF 33, 42, 43. Studies of T4P‐related T2SS, such as the T2SS of *V. cholerae*,* Erwinia chrysanthemi*, and *Xanthomonas campestris*, also revealed interactions between traffic ATPases and PilM homologs 30, 44, 45. Therefore, the PilMNO complex is a plausible candidate to interact with the ATPase PilF, thereby conveying the energy provided by ATP hydrolysis to the assembly of the DNA translocator pseudopilus and T4P.

In this study, we report on the interaction of PilM, PilMN, and PilMNO with the traffic ATPase PilF of the natural transformation system of *T. thermophilus*. Furthermore, PilMN and PilMNO complexes were found to stimulate PilF‐mediated ATPase activity. Mutant studies revealed that Lys‐26 is important for ATP binding of PilM but not required for interaction with and stimulation of PilF, whereas the GSPIIC domain of PilF is essential for the PilMN‐mediated stimulation of PilF. We propose that PilM interacts with the motor ATPase PilF, thereby functioning as ATPase‐pseudopilin/pilin coupling protein in the DNA translocator and the T4P of *T. thermophilus*.

## Results

### Isolation of PilM, PilN, PilMN, and PilMNO

**Figure 1:**
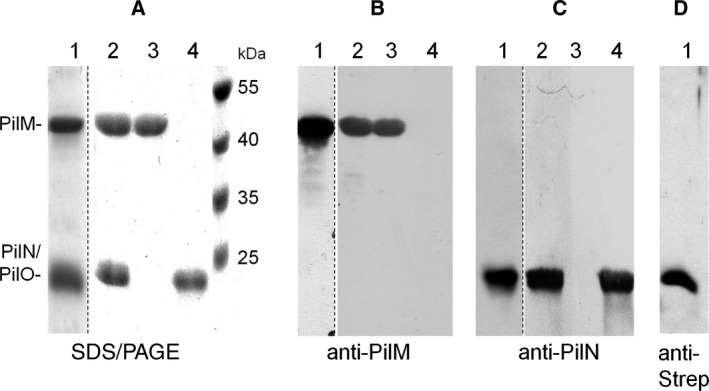
Purification of PilMNO, PilMN, PilM, and PilN. His_6_‐PilMNO‐Strep (lane 1), His_6_‐PilMN‐Strep (lane 2), His_6_‐PilM (lane 3), and PilN‐Strep (lane 4) were purified from *Escherichia coli* BL21 (DE3) cells as described in Experimental procedures. PilMNO, PilMN, and PilN were isolated from membranes, whereas PilM was isolated from soluble fractions. To separate His_6_‐PilM (44 kDa), PilN (23 kDa), and PilO‐Strep (22.5 kDa), 4–20% SDS/PAGE was performed (lane 1). All other protein preparations were separated by 14% SDS/PAGE (A). The identity of the proteins was confirmed by western blot using polyclonal antisera against PilM (B) and PilN (C) or using Strep‐Tactin HRP conjugate to detect PilO‐Strep (D). Dotted lines indicate excised lanes from different but comparable gels or blots.

To study *in vitro* interactions of the *T. thermophilus* PilMN and PilMNO complex with the traffic ATPase PilF, we engineered pET28a‐based expression plasmids encoding PilN‐Strep, His_6_‐PilMN‐Strep, or His_6_‐PilMNO‐Strep and expressed the genes in *Escherichia coli* BL21 (DE3). After cell disruption, we separated cleared lysates into membrane and soluble fractions. We found that in the strain expressing His_6_‐*pilMN*‐Strep, His_6_‐PilM was present in both fractions, whereas PilN‐Strep was only detectable in membranes. The soluble His_6_‐PilM was purified by nickel‐NTA affinity chromatography (Fig. 1A,B, lane 3). The membrane‐associated His_6_‐PilM was found to interact with PilN‐Strep as we were able to copurify the two proteins by Strep‐Tactin chromatography after solubilization of membranes with n‐dodecyl β‐d‐maltopyranoside (DDM) (Fig. 1A–C, lane 2). Membranes of the strains producing PilN‐Strep or coproducing His_6_‐PilMNO‐Strep were also treated with DDM, and the solubilized proteins were purified by Strep‐Tactin chromatography. SDS/PAGE and western blot analyses of the purified products (Fig. 1A–D, lanes 1 and 4) revealed that PilM copurified with PilN and PilO‐Strep, indicating that PilMNO forms complexes.

**Figure 2:**
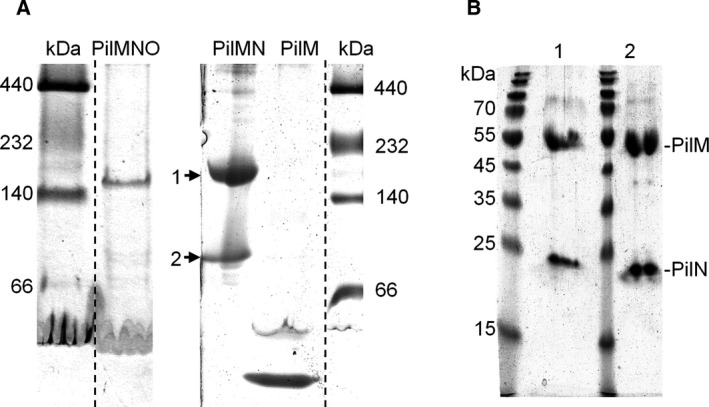
Complex formation of His_6_‐PilMNO‐Strep, His_6_‐PilMN‐Strep, and His_6_‐PilM. Complex formation was analyzed by 5–20% clear‐native PAGE (A). His_6_‐PilMN‐Strep formed two different complexes indicated with arrows. Native gels were stained with InstantBlue (Expedeon, Cambridge, UK). The protein bands corresponding to the ~ 195 kDa (1) and ~ 80 kDa (2) His_6_‐PilMN‐Strep complexes were cut out from the gel, incubated in Sol‐buffer (2% (w/v) SDS, 60 mm Na_2_CO_3_, 0.67% (v/v) 2‐mercaptoethanol) and loaded onto a 14% SDS gel. The proteins were stained with Coomassie blue (B). The dotted lines indicate excised lanes from the same gels, respectively.

Complex formation of purified His_6_‐PilMN‐Strep, His_6_‐PilMNO‐Strep, and His_6_‐PilM was further analyzed by clear‐native PAGE. His_6_‐PilMN‐Strep migrated as complexes corresponding to ~ 80 and ~ 195 kDa (Fig. 2A, arrows). SDS/PAGE of these two protein bands showed that both contain PilM and PilN (Fig. 2B). Even though native PAGE is a relatively crude method to determine the molecular mass of protein complexes, these findings demonstrate that purified, solubilized His_6_‐PilM‐PilN‐Strep formed two different oligomeric states and suggest a stoichiometry of approximately 1 : 1 and 3 : 3 for the respective oligomers. This differs from previous findings by Karuppiah *et al*. 35, where a 2 : 2 stoichiometry of PilM : PilN was found. This might be due to the use of different expression strains and differences in the purification protocols. The His_6_‐PilMNO‐Strep complex corresponds to ~ 160 kDa (Fig. 2A). This is in accordance with the 2 : 2 : 2 stoichiometry of PilM : PilN : PilO previously reported by Karuppiah *et al*. 35. His_6_‐PilM migrated as a single band corresponding to a protein of ~ 44 kDa, indicating that the soluble PilM is a monomer.

### PilM pulls down PilF

**Figure 3:**
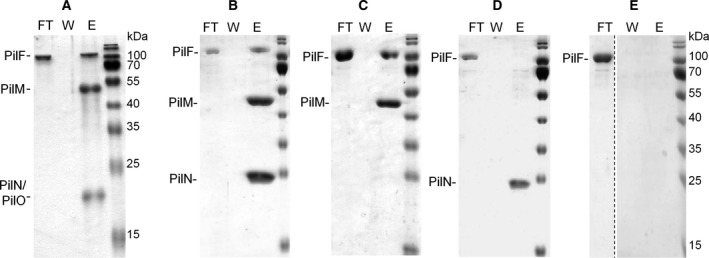
PilMNO, PilMN, and PilM pull down PilF. His_6_‐PilMNO‐Strep (A), His_6_‐PilMN‐Strep (B), Strep‐PilM (C), and PilN‐Strep (D) were bound to a Strep‐Tactin column and incubated with purified His_6_‐PilF hexamers. As a negative control, His_6_‐PilF was incubated on an empty Strep‐Tactin column (E). After washing with one column volume buffer for ten times, proteins were eluted. Flow‐through (FT) and fivefold concentrates of the tenth wash fraction (W) and eluate (E) were separated by SDS/PAGE (14% gel for A, 12% for B–E) and stained with Coomassie blue. The dotted line indicates an excised lane from the same gel.

We then examined whether the PilMN and PilMNO complexes interact with the traffic ATPase PilF. Therefore, His_6_‐PilF was heterologously produced in *E. coli* and subjected to nickel affinity chromatography followed by size exclusion chromatography as described previously 25. The purified PilF migrated in clear‐native PAGE as a single > 669‐kDa complex which corresponds to PilF hexamers (data not shown). To analyze the interaction between PilMN or PilMNO complexes with the PilF hexamers, His_6_‐PilM‐PilN‐Strep or His_6_‐PilM‐PilN‐PilO‐Strep complexes were bound to a Strep‐Tactin column, co‐incubated with excess of purified His_6_‐PilF complexes, followed by washing and elution. These studies revealed that PilF co‐eluted with both complexes (Fig. 3A,B), whereas it did not bind to the Strep‐Tactin column when applied alone (Fig. 3E). Together, these data suggest that PilF binds to PilMN and PilMNO complexes.

Next, we examined whether PilF interacts with PilM or PilN alone. Therefore, PilN‐Strep was bound to the Strep‐Tactin column followed by affinity chromatography with His_6_‐PilF complexes. No co‐elution of PilN and PilF was detected (Fig. 3D), indicating that PilN does not bind PilF. To analyze the binding of PilF to PilM, N‐terminally Strep‐tagged PilM was purified from *E. coli* via Strep‐Tactin affinity chromatography. For interaction studies, Strep‐PilM was bound to a Strep‐Tactin column followed by co‐incubation with His_6_‐PilF. PilF co‐eluted with PilM (Fig. 3C). Taken together, these results suggest that PilF interacts with the PilMNO inner membrane complex by binding to PilM.

### The PilMN complex stimulates PilF ATPase activity

**Figure 4:**
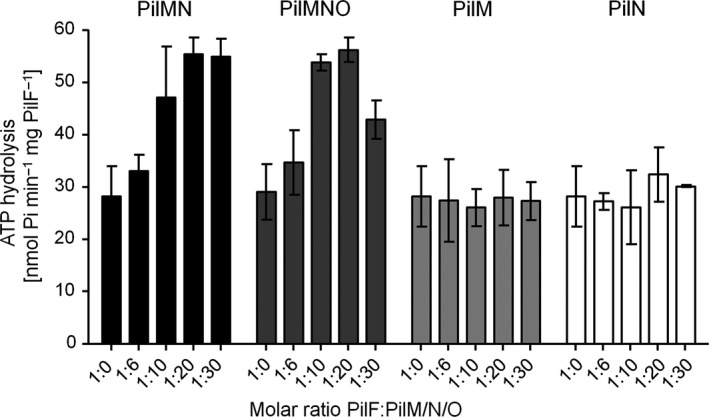
PilMN and PilMNO complexes stimulate PilF ATPase activity. ATP hydrolysis activity of PilF was measured by incubating 66.7 pmol PilF hexamer with increasing amounts of PilMN (black bars), PilMNO (dark gray bars), PilM (light gray bars), or PilN (white bars). Release of phosphate was measured over 15 min. Shown are means ± empirical standard deviations of at least three independent measurements.

Next, we addressed the question whether interaction of PilM, PilMN, and PilMNO complexes with PilF affects ATPase activity of PilF. Therefore, we mixed different amounts of purified PilMN, PilMNO, PilM, or PilN with PilF and analyzed PilF‐catalyzed ATP hydrolysis (Fig. 4). Incubation of PilF with the PilMN complex led to stimulation of ATPase activity of up to ~ 2‐fold at a ratio of 1 : 20 (PilF complex : PilMN complex). The same was observed when PilMNO was added to PilF hexamers. The addition of PilM or PilN alone had no effect. No ATPase activity was detected with PilMN or PilMNO complexes in the absence of PilF. Taken together, these data lead to the conclusion that interaction of PilM and PilF only leads to increased ATPase activity when PilM is bound to PilN.

### Lys‐26 is important for ATP binding of PilM

**Figure 5:**
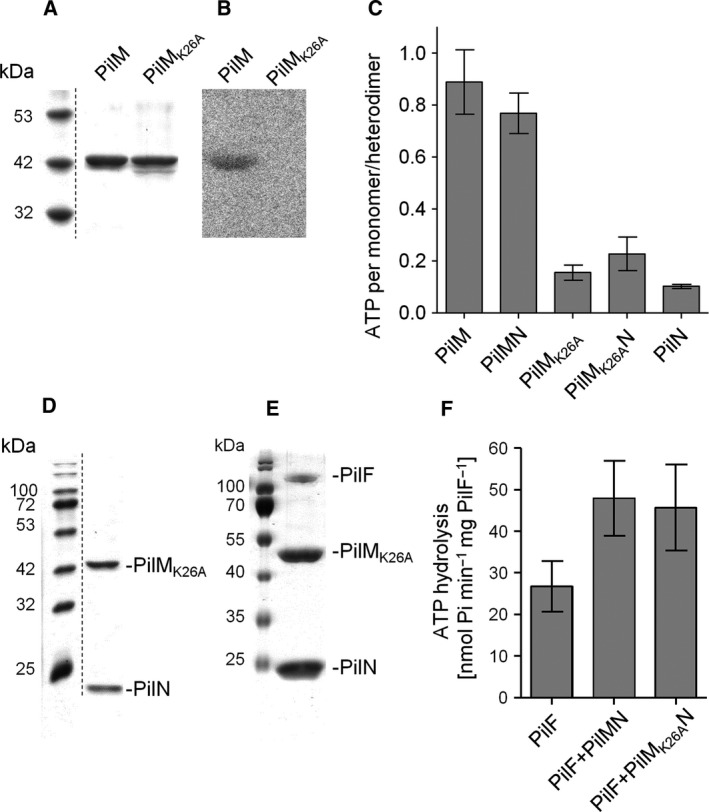
PilM_K26A_ does not bind ATP but stimulates PilF ATPase activity. Two micrograms purified His_6_‐PilM and His‐PilM_K26A_ was separated by SDS/PAGE. The dotted line indicates an excised lane from the same gel (A). ATP binding by PilM variants was analyzed by incubating 2 μg of purified protein with [α‐^32^P]‐ATP and photo cross‐linking. Proteins were precipitated and separated by SDS/PAGE. Radioactivity was detected on a storage phosphor screen (B). To analyze ATP content after purification, proteins were precipitated with TCA on ice. Na‐TES buffer and K_2_CO_3_ were added to adjust pH. After centrifugation, the ATP content of the supernatant was determined using firefly lantern extract as described in Experimental procedures (C). His‐PilM_K26A_ and PilN‐Strep were coproduced in *Escherichia coli* and copurified from solubilized membranes via Strep‐Tactin affinity chromatography. One microgram of purified protein was separated in 14% SDS/PAGE. The dotted line indicates an excised lane from the same gel (D). His_6_‐PilM_K26A_N‐Strep pulled down PilF when co‐incubated on a Strep‐Tactin column as described in Fig. 2 (E). PilM_K26A_N stimulates PilF ATPase activity similar to wild‐type PilMN. PilF hexameric complexes were mixed with PilMN or PilM_K26A_N in a molar ratio of 1 : 20 for ATPase assays (F). Shown are means ± SD of at least three independent measurements.

Structural analyses of a PilM‐PilN_1–15_ complex by Karuppiah and Derrick 34 revealed that PilM binds ATP and that Lys‐26, which is highly conserved in PilM homologs, is located adjacent to the α‐ and β‐phosphates of bound ATP. To examine the role of Lys‐26 in ATP binding of PilM, we generated a His_6_‐PilM_K26A_‐PilN‐Strep variant via site‐directed mutagenesis, followed by overproduction in *E. coli* BL21 (DE3), separation of the soluble fraction from membranes, purification of the PilM_K26A_ variant via nickel‐NTA affinity chromatography from the soluble fraction, and purification of PilM_K26A_N via Strep‐Tactin affinity chromatography from solubilized membranes. This led to pure His_6_‐PilM_K26A_ protein and His_6_‐PilMK26AN‐Strep complexes, respectively (Fig. 5A,D). To examine ATP binding of PilM and the role of Lys‐26 in ATP binding, we incubated purified His_6_‐PilM and His_6_‐PilM_K26A_ with [α‐P^32^]‐ATP and performed photo cross‐linking followed by autoradiography (Fig. 5B). This led to the detection of ATP bound to the wild‐type PilM protein, whereas no ATP was found in the PilM_K26A_ variant. This suggests that Lys‐26 plays an important role in ATP binding of PilM.

To verify this finding, we analyzed the ATP content of purified wild‐type PilM and PilMN complex and of purified PilM_K26A_ and PilM_K26A_N variants without the addition of further ATP. Therefore, purified proteins were precipitated with TCA and the supernatants were subjected to luciferase assays (Fig. 5C). Purified PilN served as negative control. We found that PilM wild‐type protein contains 0.89 ± 0.35 ATP per PilM monomer after purification and PilMN wild‐type complexes had 0.77 ± 0.17 ATP per heterodimer. In purified PilN, 0.10 ± 0.02 ATP per protein was detected. The amounts of ATP in PilM_K26A_ and PilMK26AN were comparable to the amounts of ATP in the PilN negative control. Taken together, these data show that Lys‐26 is important for ATP binding of PilM.

### PilM_K26A_N binds PilF and stimulates ATPase activity

To examine whether ATP binding to PilM is important for the binding of PilF to the PilMN complex, purified PilM_K26A_N complex was immobilized on an Step‐Tactin column followed by incubation and chromatography with His_6_‐PilF. These studies revealed that His_6_‐PilF still co‐eluted with the PilM_K26A_N complex (Fig. 5E). We further addressed the question whether the purified PilM_K26A_N complex stimulates the ATPase activity of the PilF hexamer. Therefore, purified His_6_‐PilF hexamer was mixed with PilM_K26A_N complex in a ratio of 1 : 20 followed by ATPase assays. Still, a ~ 2‐fold activation of the ATPase activity was observed (Fig. 5F). This leads to the conclusion that ATP binding of PilM is not important for *in vitro* stimulation of PilF by the PilMN complex.

### The GSPIIC domain is important for PilMN‐mediated stimulation

**Figure 6:**
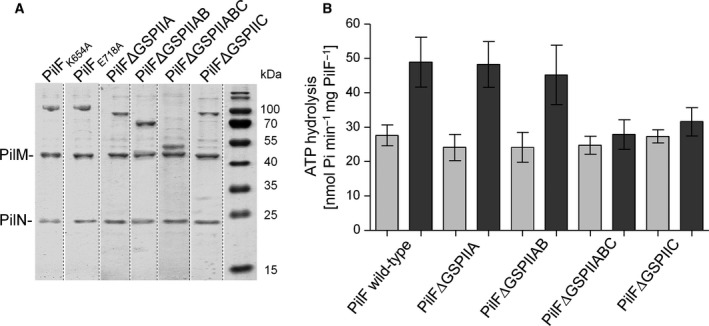
The PilF GSPIIC domain is necessary for PilMN‐mediated stimulation but not for interaction. Co‐elution of PilMN with PilF_K654A_, PilF_E718A_ and PilFΔGSPII variants. Dotted lines indicate excised lanes from identical gels (A). ATPase assay of PilFΔGSPII variants alone (light bars) and in presence of PilMN in a ratio of 1 : 20 (dark bars) as described above (B). Bars represent means ± SD of at least three independent measurements.

For the traffic ATPase XpsE of the T2SS of *X. campestris*, it has been described that ATP binding is necessary for interaction with the N terminus of EpsL, which is a structural homolog to PilM 46. We recently generated PilF variants with defects in ATP binding (PilF_K654A_) or ATP hydrolysis (PilF_E718A_), both of which did not exhibit any ATPase activity 47. Both variants still formed high molecular mass complexes. PilF_K654A_ migrated slightly slower in native PAGE than wild‐type PilF, which might be due to differences in tertiary and/or quaternary structure. We co‐incubated the PilF_K654A_ and PilF_E718A_ variants with Strep‐Tactin‐bound PilMN complexes. PilF_K654A_ as well as PilF_E718A_ co‐eluted with PilMN (Fig. 6A), indicating that ATP binding or hydrolysis by PilF is not required for co‐elution with the PilMN complex.

Traffic ATPases involved in T4P assembly and in T2SS harbor a conserved fold‐designated GSPII domain 29, 30. For the T2SS traffic ATPases XpsE and EpsE from *X. campestris* and *V. cholerae,* it has been shown that they interact with the structural PilM homologs XpsL and EpsL, respectively, via their GSPII domain containing N termini 29, 30, 48. PilF of *T. thermophilus* differs from all known traffic ATPases as it contains three N‐terminal GSPII domains (GSPIIA, B and C) 25. Thus, the question arose whether PilF interacts with PilM via one or multiple GSPII domains leading to stimulation of ATPase activity. To address this question, we used a set of PilF variants depleted of GSPII domains. PilF variants with deletions of the first two or all three GSPII domains formed altered and/or unstable PilF complexes, but all variants exhibited wild‐type ATPase activity 47. We found that PilF variants missing the first or the first two GSPII domains were still stimulated by addition of PilMN. However, no significant stimulation was observed for PilFΔGSPIIABC or PilFΔGSPIIC (Fig. 6B). Our recent studies revealed that PilFΔGSPIIABC complexes were rather unstable, but PilFΔGSPIIC formed stable hexameric complexes 47. Thus, the GSPIIC domain is important for PilMN‐mediated stimulation of ATPase activity of PilF complexes. To test whether this is due to abolished interaction between PilM and the PilF variants, we again bound PilMN to Strep‐Tactin columns and co‐incubated with the different PilF variants. These studies confirmed that PilFΔGSPIIA and PilFΔGSPIIAB interact with PilMN. Interestingly, also PilFΔGSPIIC and PilFΔGSPIIABC co‐eluted with PilMN, indicating that none of the GSPII domains is essential for interaction with PilMN and implying that PilMN binds primarily to the C‐terminal domain of PilF (Fig. 6A).

## Discussion

Previous analyses of the DNA translocator in *T. thermophilus* revealed that PilM, PilN, and PilO are associated with the inner membrane and therefore are good candidates to be part of the inner membrane platform 18. The inner membrane association of PilN and PilO is consistent with the presence of single transmembrane helices. However, the inner membrane association of PilM was unexpected at that time since PilM did not contain any transmembrane domains. Therefore, the association of PilM with the inner membrane must be due to interaction of PilM with membrane integral proteins of the inner membrane platform. This was supported by structural analyses of cocrystals of PilM and a synthetic PilN peptide 34. These analyses revealed that the first eight residues of PilN bind to PilM in a narrow channel between two subdomains. However, studies that use only small peptides instead of the native protein may not be unambiguous since the missing majority of the protein may sterically hinder interaction of these amino acids with the target protein.

Heterologous expression of *T. thermophilus* PilM confirmed the soluble character of PilM, whereas heterologous co‐expression of PilM and PilN led to membrane association of PilM 34, 35. The same was found in heterologous co‐expression analyses of PilM, PilN, and PilO. These data confirm that PilM interacts with PilN and that PilM, N, and O form a complex.

Despite intensive studies on the interaction of inner membrane platform proteins of the DNA translocator and the T4P system in *T. thermophilus,* the key question how exactly the biogenesis of the T4P and the DNA translocator starting from the inner membrane is powered is still open. It seemed likely that the traffic ATPase PilF, which plays a dual role in the DNA translocator and T4P of *T. thermophilus* 9, 11, interacts with the inner membrane platform, thereby conveying the energy provided by ATP hydrolysis to both systems. Here, we have demonstrated that the traffic ATPase PilF interacts with the PilMNO inner membrane platform. Our finding that PilM, but not PilN pulls down PilF suggests that the interaction between PilF and the PilMNO complex is mediated by PilM. In this context, it is important to notice that interaction between traffic ATPases and PilM orthologs is not consistent throughout all T4P systems. For the traffic ATPase PilF in *N. meningitidis,* no interaction between the two proteins was detectable in bacterial two‐hybrid assays 36, whereas for the T4P traffic ATPases PilB of *M. xanthus* and of *P. aeruginosa* and for heterologously produced PilF of *N. meningitidis,* direct interaction with PilM has been shown 33, 42, 43. In the first two systems, PilB was also shown to interact with the integral membrane protein PilC 33, 49. In *M. xanthus,* this interaction even led to stimulation of PilB ATPase activity. A potential interaction of the *T. thermophilus* PilC protein with the traffic ATPase PilF will be the subject of future studies. Taken together, our data are consistent with the hypothesis that PilM is a coupling protein connecting the ATPase with the inner membrane subcomplex triggering the assembly of the T4P and a DNA translocator pseudopilus.

**Figure 7:**
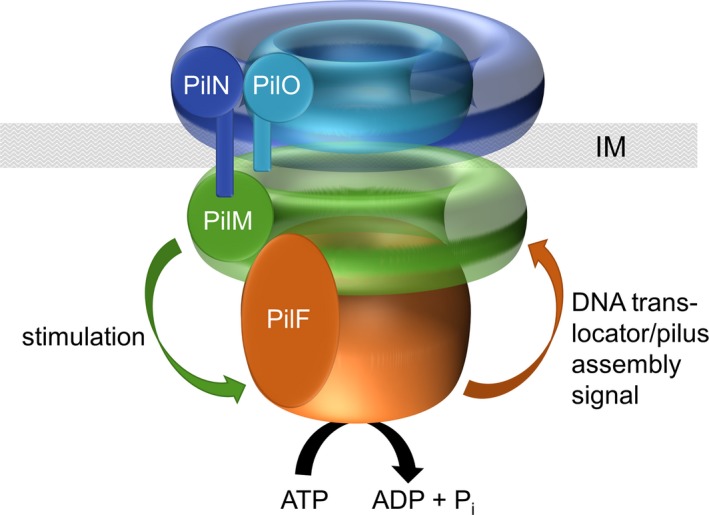
Model of the inner membrane platform interacting with the traffic ATPase PilF. PilN and PilO interact at the periplasmic face of the inner membrane (IM). The cytoplasmic N terminus of PilN interacts with the cytoplasmic PilM. PilM, when bound to PilN, stimulates activity of the soluble ATPase PilF. The energy provided by hydrolysis of ATP is then transmitted from PilF via the inner membrane platform comprising PilM, PilN, and PilO to further components of the DNA translocator/T4P machinery leading to DNA uptake and T4P assembly.

Recently, we reported on the first *in situ* structure of the T4P machinery of *T. thermophilus* in the open and closed states 14. A central channel comprising of secretin subunits forming several ring‐shaped domains was found to be anchored in the outer membrane. Furthermore, two protein densities (P1 and P2) were detected in the periplasm outside of the peptidoglycan layer and one protein density (C1) was found close to the outside of the inner membrane. In the open state of the machinery, an extra protein density was detected in the cytoplasm. This *in situ* structure together with the data presented here leads to the following model of the inner membrane assembly platform of T4P and the DNA translocator of *T. thermophilus* (Fig. 7): We propose that the membrane‐anchored PilN and PilO proteins form the protein density C1. The N terminus of PilN interacts with the cytoplasmic protein PilM. PilM binds to PilF which could be the extra protein density in the cytoplasm detected in the open state. The binding of PilF to the PilMNO complex leads to stimulation of PilF ATPase activity. Conformational changes in PilF due to ATP hydrolysis 27, 28, 50 are then transmitted via PilM to PilN and PilO which ultimately allows DNA uptake and T4P assembly.

Functional interaction of the coupling protein PilM with PilF seems to require the presence of PilN as only in the presence of both, PilM and PilN, *in vitro* ATPase activity of PilF is stimulated. This may result from an alteration of the ATP‐binding site in PilM and/or rearrangement of PilM domains upon binding of PilN as suggested by structural analysis of PilM from *P. aeruginosa* 43.

The stimulation of a DNA translocator traffic ATPase by the inner membrane platform proteins PilMN has not been reported before. However, this has been observed for the traffic ATPases in T2SSs of *V. cholerae* and *X. campestris*. Structural studies have shown that the T2SS ATPase EpsE (XpsE) interacts with EpsL (XpsL), a natural genetic fusion of PilM and PilN 30, 46. Analogously to our finding, this interaction also led to ~ 2‐fold stimulation of EpsE (XpsE)‐mediated ATPase activity 46, 51. The stimulation of EpsE of *V. cholerae* was further increased when acidic phospholipids were added additionally (stimulation of up to 130‐fold). Stimulation of ATPases by membrane lipids has also been described for the archaeal flagellum assembly ATPase FlaI of *Sulfolobus acidocaldarius*, the flagellar export ATPase FliI of *Salmonella typhimurium* and the secretion ATPase SecA of *E. coli* 52, 53, 54. The possible interaction of *T. thermophilus* PilF with membrane phospholipids will be studied in the future.

The T2SS ATPases have been shown to interact with EpsL via their N terminus which comprises a GSPII fold (αββαβα) 29, 30. The N terminus of *T. thermophilus* contains three GSPII domains. Recently, we analyzed truncated PilF variants devoid of one or more GSPII domains and could show that variants devoid of the first two or of all three GSPII domains show altered complex formation 47. For some traffic ATPases, it has been shown that their oligomers exhibit higher activities than the monomers 32, 33, 55, 56. Crystal structures of the hexameric T4P assembly ATPase of *Geobacter metallireducens* and of the C‐terminal domains of *T. thermophilus* PilF suggest a coordinated cycle of ATP binding and hydrolysis throughout the hexamer, indicating an interplay of the protomers 50, 57. However, the recently published structure of the full‐length PilF suggests that shifting of PilF domains relative to each other upon ATP binding and hydrolysis might play a more important role than changes in the interactions with adjacent protomers 27. This is also in agreement with our finding that the PilF variants forming altered complexes, such as PilFΔGSPIIAB or PilFΔGSPIIABC, still displayed wild‐type ATPase activities 47. Here, we show that these variants still interact with PilM. PilF variants devoid of GSPIIC or GSPIIABC could not be stimulated by PilMN, indicating that the GSPIIC domain is important for transferring a signal generated by PilM binding to the C‐terminal ATPase domain of PilF. This is also consistent with the finding that *T. thermophilus* mutants expressing these truncated *pilF* genes are defective in T4P biogenesis and twitching motility 47. Interestingly, the mutant expressing *pilFΔGSPIIC* was hypertransformable, indicating that the stimulatory effect of PilMN on PilF‐mediated ATPase activity is not necessary for natural transformation. To address this finding, the dynamic interactions between PilF and other components playing a single role in the DNA translocator, such as the minor pilins PilA1, PilA2, or PilA3 or ComZ should be studied in the future.

## Experimental procedures

### Organisms and cultivation

*Escherichia coli* DH5α, BL21 (DE3) and T7 Express cells harboring pET28a plasmids (New England Biolabs, Ipswich, MA, USA) were grown at 37 °C in LB medium or 2 × YT medium containing 20 μg·mL^−1^ kanamycin.

### Cloning, overexpression, and purification of PilN, PilM, PilMN, and PilMNO

PilN, PilM, and PilMN were produced using the plasmid pET28a system. For amplification of *pilN*‐Strep, the primer pair pilN‐for and pilN‐Strep‐rev was used (Table 1). The primer pair pilM‐for and pilN‐Strep‐rev was used to amplify the overlapping genes *pilM* and *pilN* from genomic DNA of *T. thermophilus* HB27. PCR products were cloned into pET28a using NcoI and NotI for *pilN* or NdeI and NotI for *pilMN* resulting in plasmids listed in Table 1. These plasmids were transformed into *E. coli* BL21 (DE3), and gene overexpression was induced at an OD_600_ of ~ 1 by adding IPTG to a final concentration of 1 mm. Cells were harvested after ~ 4 h growth at 37 °C and washed with MN buffer [50 mm Tris/HCl, pH 7.5, 200 mm NaCl, 5 mm MgCl_2_, 5% (v/v) glycerol].

**Table 1:** Primers used in this study to generate the plasmids and resulting protein variants indicated

Primer	Sequence 5′–3′	Resulting plasmids	Protein(s)
pilN‐for	AGGACCATGGTGATTAGGCTGAACCTTCTCCCCAAAAACC	pET28a‐PilN	PilN‐Strep
pilN‐Strep‐rev	ATTAGCGGCCGC*TTATTTTTCGAACTGCGGGTGGCTCCAAGCGCT*GCGAGCACCGCTTTCACCCCC	pET28a‐PilN, pET28a‐PilMN	PilN‐Strep, His_6_‐PilMN‐Strep
pilM‐for	GCCACCATATGTTCAAAAGCCTTAGCCAGCTCTTCC	pET28a‐PilMN	His_6_‐PilM, His_6_‐PilMN‐Strep
pilM‐His‐for	GCCACATATG*CATCACCACCATCACCATCACTTC*AAAAGCCTTAGCCAGCTC	pET28a‐PilMNO	His_6_‐PilMNO‐Strep
pilO‐Strep‐rev	ATATGCGGCCGC*TCATTTTTCGAACTGCGGGTGGCTCCAGGCGCT*TGGGGTGCTCCCTCCCGTTTCC		
pilM‐Strep‐for	TGAACCATGG*CTTGGAGCCACCCGCAGTTCGAAAAATCCGCTTTCA*AAAGCCTTAGCCAGCTCTTC	pET28a‐Strep‐PilM	Strep‐PilM
pilM‐rev	ATTAGCGGCCGCCTAATCAAGGGGCTCCACCCCCCTC		
pilM‐K26A‐for	GCACTCGTGGAGGTGTCCGGGAACC	pET28a‐Strep‐PilM_K26A_, pET28a‐PilM_K26A_N	Strep‐PilM_K26A_, His_6_‐PilM_K26A_, His_6_‐PilM_K26A_N‐Strep
pilM‐K26A‐rev	CAGGGCGGAGGCCCCGATCTCCAAG		

Restriction enzyme sites are underlined; sequences for tags are in italics.

Cells were resuspended in MN buffer containing 1 mg·mL^−1^ DNase I and 1 mm PMSF and disrupted by two passages through the French pressure cell (16 000 psi). Cell debris was removed by centrifugation at 11 000 ***g***, and membranes were separated from soluble parts by ultracentrifugation at 244 000 ***g***, 4 °C for 50 min. For purification of PilN and for copurification of PilMN, membranes were washed with MN buffer, centrifuged at 244 000 ***g***, 4 °C for 50 min, and then solubilized in MN buffer containing 1% (w/v) DDM for 1 h at 4 °C. Samples were again centrifuged at 244 000 ***g***, 4 °C for 45 min to remove insoluble membrane fractions. Supernatants were loaded on a Strep‐Tactin Superflow High capacity column (IBA, Göttingen, Germany). Gravity‐flow purification was performed according to the manufacturer's instructions using MN buffer containing 0.02% DDM and 2.5 mm d‐desthiobiotin. Eluates were concentrated using centrifugal concentrator tubes with a molecular mass cutoff of 10 kDa (PilN) or of 30 kDa (PilMN).

His‐PilM was purified from soluble fractions of cells expressing *pilMN*. Supernatants from ultracentrifugation were again centrifuged at 244 000 ***g***, 4 °C for 50 min to remove the membranes and then incubated with nickel‐NTA agarose at 4 °C for 1 h. Samples were then filled into a gravity‐flow column, washed with 10 column volumes MN buffer with 35 mm imidazole. His_6_‐PilM was eluted in 2.5 column volumes MN buffer with 350 mm imidazole. Purified PilM was concentrated with a molecular mass cutoff of 10 kDa.

For co‐expression of *pilMNO*, the three genes were amplified using primers pilM‐His‐for and pilO‐Strep‐rev and cloned into pET28a using NdeI and NotI restriction sites. The resulting plasmid was transferred into T7 Express cells (New England Biolabs) for overproduction of His_6_‐PilMNO‐Strep. Cells were grown in 2 × YT medium at 37 °C until an OD_600_ of ~ 1 was reached. Then, IPTG was added to a final concentration of 0.1 mm followed by overnight incubation at 16 °C (as described in Ref. 35). Cells were harvested by centrifugation at 7025 ***g*** and washed with MNO buffer [25 mm Tris/HCl, pH 8.0, 100 mm NaCl, 10 mm MgCl_2_, 5% (v/v) glycerol]. Cell disruption, isolation of membranes, solubilization, and Strep‐Tactin affinity chromatography were conducted as described above using MNO buffer. PilMNO containing eluates were concentrated using a 100‐kDa cutoff concentrator and were then loaded on a Superdex 200 (10/300) gel filtration column (GE Healthcare, Chicago, IL, USA) equilibrated with MNO buffer containing 0.02% DDM. Peak fractions were pooled and again concentrated.

### Generation of PilM_K26A_

For generation of PilM_K26A_ variants, we followed the Phusion site‐directed mutagenesis protocol (New England Biolabs) using pET28a‐Strep‐PilM and pET28a‐PilMN as templates and primers pilM‐K26A‐for and pilM‐K26A‐rev. Strep‐PilM_K26A_, His‐PilM_K26A_, and His‐PilM_K26A_‐PilN‐Strep were purified as described for the wild‐type variants.

### Column‐based interaction studies

PilF complexes were produced and purified as described previously 26. Generation and purification of PilF variants PilFΔGSPIIA, PilFΔGSPIIAB, PilFΔGSPIIABC, PilFΔGSPIIC, PilF_K654A_, and PilF_E718A_ have also been described previously 47.

To analyze the interaction between PilF and components of the inner membrane subcomplex, PilM, PilN, PilMN, or PilMNO was co‐incubated with His_6_‐PilF on a Strep‐Tactin column. For this, we generated Strep‐PilM by first amplifying *pilM* from genomic DNA using primers pilM‐Strep‐for and pilM‐rev (Table 1). The PCR product and pET28a were treated with NcoI and NotI and ligated. The Strep‐PilM expression plasmid was transferred into *E. coli* BL21 (DE3), and protein production and cell disruption were performed as described above. Strep‐PilM was purified from cell‐free lysate via a Strep‐Tactin Superflow High capacity column (IBA) following the manufacturer's instructions.

For *in vitro* interaction studies, the bait proteins (Strep‐PilM, PilN‐Strep, His_6_‐PilMN‐Strep, His_6_‐PilMNO‐Strep, or His_6_‐PilM_K26A_N‐Strep) were purified from ~ 15 g *E. coli* cells as described above up to the point where proteins were bound to 1 mL of Strep‐Tactin Superflow high‐capacity material. Columns were washed with 5 mL MN buffer (incl. 0.02% DDM). Then, 500 μg purified PilF in 1 mL bicine buffer (100 mm bicine, 200 mm NaCl, 0.02% DDM, pH 8.5) was co‐incubated on column for 15 min at room temperature. After washing with 1 mL MN buffer for 10 times (incl. 0.02% DDM), proteins were eluted with 3 mL elution buffer (MN buffer incl. 0.02% DDM and 2.5 mm d‐desthiobiotin). Wash fractions and eluates were fivefold concentrated using a centrifugal concentrator tube with a molecular mass cutoff of 10 kDa. Flow‐through, the tenth wash fraction and eluates were analyzed by SDS/PAGE. As a negative control, 500 μg of purified PilF was loaded on an empty Strep‐Tactin column. The column was washed and eluted as above. No protein could be detected in the eluate fraction.

### PAGE and western blot analyses

For western blot analyses, 3 μg of protein was separated in denaturing SDS/PAGE using 12% or 14% gels 58. Purified PilMNO was analyzed by separating 1 μg protein in a 4–20% gradient SDS gel (Nippon Genetics Europe, Düren, Germany). Immunodetection was performed using polyclonal antiserum against PilM (dilution 1 : 5000) or PilN (dilution 1 : 5000) as described previously 18. For detection of PilO‐Strep, we used Strep‐Tactin HRP conjugate (IBA) according to the manufacturer's instructions. PilM, PilMN and PilMNO complexes were analyzed by 5–20% clear‐native PAGE 59, whereas the PilF complex was analyzed by 3–15% clear‐native PAGE. To visualize the individual proteins of the His_6_‐PilMN‐Strep complexes, bands of the respective native polyacrylamide gel were cut out and incubated in Sol‐buffer [2% (w/v) SDS, 60 mm Na_2_CO_3_, 0.67% (v/v) 2‐mercaptoethanol] for 20 min at room temperature. The protein bands were loaded onto a 14% SDS gel and overlaid with 0.5% (w/v) agarose dissolved in SDS running buffer [0.1% (w/v) SDS, 25 mm Tris, 192 mm glycine, pH 8.5 adjusted with HCl]. After polymerization of the agarose, SDS/PAGE was performed.

### ATPase assays

ATP hydrolysis was analyzed using malachite green assay as described by Camberg and Sandkvist 55. Two hundred microliters of reaction mixture containing 40 μg PilF (corresponding to 66.7 pmol hexamer), 5 mm MgCl_2_ and 3 mm ATP in TMC buffer [50 mm MOPS, 50 mm Tris, 50 mm CHES, 50 mm glycine, 50% (v/v) glycerol, 150 mm KCl, pH 9.5 adjusted with KOH] was incubated at 68 °C for 15 min, which are conditions under which phosphate release was linear over time and in dependence of the PilF concentration. Forty microliters of samples were taken every 5 min, proteins were precipitated by the addition of 8 μL 30% TCA, and phosphate was determined in duplicate. Malachite green reagent (800 μL containing 1.05% ammonium molybdate, 0.034% malachite green hydrochloride, 0.1% Triton X‐100, 1 N HCl) was incubated with 40 μL reaction mixture. Then, 100 μL of 34% sodium citrate was added and samples were incubated for 20 min at room temperature. Absorbance at 650 nm was measured and compared to a phosphate standard curve.

To analyze stimulation of PilF, different amounts of purified PilMNO, PilMN, PilM, or PilN were added to PilF and volumes were equalized by adding MN buffer, so that the DDM concentration in the 200 μL reaction mixture always exceeded the critical micelle concentration. Reactions were started by addition of ATP. ATPase activity of PilMNO, PilMN, PilM, and PilN was tested using 2 nmol of each protein or protein complex, respectively.

### ATP binding

ATP binding of PilM variants was monitored by cross‐linking of PilM with [α‐^32^P]‐ATP as described by Babst *et al*. 60. Twenty microliters of reaction mixtures containing 2 μg of purified PilF protein and 15 μCi [α‐32P]‐ATP (> 3000 Ci·nmol^−1^) in MN buffer containing 0.02% DDM were incubated on ice for 10 min. Photo cross‐linking was carried out at ~ 5 cm distance to a UV lamp (254 nm) for 10 min on ice. Ten percent of trichloroacetic acid was added to precipitate proteins. After washing with acetone, proteins were resolved in SDS loading buffer and separated by SDS/PAGE. Radioactive signals were monitored by exposing a storage phosphor screen (GE Healthcare) to the dried gel.

To determine the ATP content in PilM and PilMN purified from *E. coli,* firefly luminescence analyses were performed. For this, defined amounts of purified PilM, PilN, or PilMN in 80 μL MN buffer were precipitated by addition of 10% TCA and 15‐min incubation on ice. After addition of 11.6 μL saturated K_2_CO_3_ solution and 18.6 μL Na‐TES (0.4 m, pH 7.4), samples were centrifuged for 10 min at 16 200 ***g*** and 4 °C. Twenty microliters of the supernatant was mixed with 250 μL sodium arsenate solution (5 mm Na_2_HAsO_4_, 4 mm MgSO_4_, 20 mm glycylglycine, pH 8) and 5 μL firefly lantern extract (Sigma‐Aldrich, St. Louis, MO, USA). Luminescence was measured for 10 s, and ATP concentrations were calculated with the help of an ATP standard.

## Conflict of interest

The authors declare no conflict of interest.

## Author contributions

KK conducted experiments, analyzed the data, and wrote the manuscript. RS conducted experiments and analyzed the data. BA supervised the project, analyzed the data, and wrote the manuscript.
